# *Microdochium majus* Isolated from Grapevine Is a Mycoparasite of *Botrytis cinerea*

**DOI:** 10.3390/jof11010031

**Published:** 2025-01-04

**Authors:** Kálmán Zoltán Váczy, Dóra Szabó, Nikolett Molnár, Tibor Kiss, Levente Kiss, Yu Pei Tan, Ádám Novák, Xénia Pálfi, Adrienn Gomba-Tóth, Zoltán Karácsony

**Affiliations:** 1 Food and Wine Research Institute, Eszterházy Károly Catholic University, Leányka utca 8/G, H-3300 Eger, Hungary; szabo.dora@uni-eszterhazy.hu (D.S.); molnar.nikolett@uni-eszterhazy.hu (N.M.); kiss2.tibor@uni-eszterhazy.hu (T.K.); levente.kiss@unisq.edu.au (L.K.); novak.adam@uni-eszterhazy.hu (Á.N.); palfi.xenia@uni-eszterhazy.hu (X.P.); gomba.toth.adrienn@uni-eszterhazy.hu (A.G.-T.); karacsony.zoltan@uni-eszterhazy.hu (Z.K.); 2 Centre for Crop Health, University of Southern Queensland, Toowoomba, QLD 4350, Australia; yupei.tan@daf.qld.gov.au

**Keywords:** biocontrol, endophyte, fungal parasite, host preference

## Abstract

The best known *Microdochium* spp. are important pathogens of small-grain cereals and/or endophytes of diverse monocot hosts. This study is the first report of *M. majus* isolated from asymptomatic grapevine tissues. It was hypothesised that this *M. majus* strain, CBS 152328, was an endophyte and an antagonist of some fungal pathogens of grapevine. Microscopic examinations revealed that this strain was a necrotrophic mycoparasite of *Botrytis cinerea*. This was demonstrated in the confrontation zones of dual cultures of *M. majus* and *B. cinerea*, and also on the surface of co-inoculated grape leaf discs and germinated wheat grains. Pathogenicity tests indicated that *M. majus* can colonise both grape leaf discs and germinated wheat, but it only damaged wheat. When co-inoculated with *B. cinerea* onto grape leaf discs, the *M. majus* strain CBS 152328 suppressed its mycohost on grape tissues and prevented leaf necrosis caused by *B. cinerea*. In addition to the parasitism, *M. majus* also showed mild antibiosis against *B. cinerea*, as well as a defence elicitor effect on grape leaf discs. This work is the first report of the mycoparasitic behaviour of *M. majus*, in addition to its first isolation from a dicot host.

## 1. Introduction

The ascomycetous genus *Microdochium* was established a century ago with the description of the type species *Microdochium pharagmitis* [1,2]. Some *Microdochium* species are saprobic, soil-borne fungi, while others are closely associated with their plant hosts, mostly monocots, as pathogens or endophytes [2]. In terms of their economic significance, *M. majus* and *M. nivale* are important cereal pathogens that cause significant yield losses in wheat and barley as part of disease complexes known as seedling blight, Fusarium head blight, Fusarium ear blight, and pink snow mould [2,3,4]. Other *Microdochium* species are known as pathogens of diverse monocots [5,6,7]. Their pathogenic lifestyle depends on the acquisition of nutrients from the infected host plant tissues [5,6,7]. *Microdochium nivale*, one of the most studied species of this genus, is known to produce a wide range of enzymes that degrade constituents of the host plants [8]. The genome of *M. nivale* was recently sequenced and revealed the genetic potential to produce mycotoxins (fumonisin, ochratoxin B, aflatoxin, and gliotoxin) in its monocot hosts [9].

Little is known about the occurrence and the possible ecological role of *Microdochium* species in dicots. Metabarcoding-based analysis of endophytic microbiota of various plants identified *Microdochium* strains in *Colobanthus quitensis* [10] and *Vitis vinifera* [11], and an isolation-based study reported *Microdochium* in *Cucurbita pepo* [12].

The relationship between *Microdochium* spp. and other fungi that co-inhabit the same plant hosts is also little understood. In some studies, *Microdochium* strains were reported as antagonists of phytopathogenic fungi. *Microdochium bolleyi*, known as an endophyte of cereals [13], and reported as a causal agent of root rot of triticale as well [6], was identified as an antagonist of *Fusarium culmorum* in *Brachypodium distachyon* and wheat hosts [13]. This fungus was also able to inhibit the damaging effects of *Bipolaris sorokiniana* and *Gaeumannomyces graminis* in barley [14,15]. While *M. bolleyi* showed antibiosis against *F. culmorum* in dual culture assays [16] and promoted the growth of inoculated barley [15], its possible biocontrol modes of action are unknown.

As part of a series of projects conducted by our laboratory to isolate diverse filamentous fungi from different grapevine samples collected in Hungarian vineyards [17,18], an isolate obtained from an asymptomatic grapevine cane sample was identified as *M. majus*. This was surprising, as *M. majus* is mainly known as a pathogen of monocots, including small-grain cereals and grasses [2,3,4]. As some *Microdochium* spp. are known as endophytes [2,13] and antagonists of some plant pathogenic fungi [14,15,16], and some endophytes isolated from grapevine may have biocontrol potential against grapevine trunk diseases [19], we hypothesised that the newly isolated *M. majus* strain may play a similar role in grapevine tissues. This study was conducted to verify this hypothesis by (a) microscopic examination of the confrontation zones between *M. majus* isolated from grapevine and *Botrytis cinerea* in dual cultures and (b) studying the interactions between *M. majus* from grapevine and *B. cinerea* on co-inoculated grape leaf discs. *Botrytis cinerea* was selected for this study because it is the causal agent of grey mould and noble rot of grapevine that is commonly found in the vineyards of the wine region where the *M. majus* strain comes from [20,21].

## 2. Materials and Methods

### 2.1. Fungal Isolates

The *M. majus* strain was isolated from a grapevine cane as follows. The sampled cane was cut into thin discs and subjected to surface sterilisation by immersing in 70% (*v/v*) ethanol for 2 min, followed by treatment in 4% (*m/v*) sodium hypochlorite for 2 min and in 70% (*v/v*) ethanol for 2 min. The samples were dried on a sterilised paper towel and cut into five pieces. The wood chips were placed on top of PDA plates amended with 10 µg/mL oxytetracycline to prevent bacterial growth. Cultures were incubated at 25 °C until fungal colonies appeared. Small portions of growing mycelia were sequentially subcultured two times on PDA plates and incubated at 21 °C in the dark to obtain clean cultures.

To identify the newly isolated fungus, DNA was extracted from fungal mycelia growing on PDA plates as described by Zhang et al. [22]. PCR reactions were carried out to amplify four loci of the isolate for subsequent Sanger sequencing. The internal transcribed spacer region (ITS) of the nrDNA was amplified with primers ITS1-f and ITS4; a fragment of the β-tubulin (*tub2*) gene with primers Btub526_F and Btub1332_R; a part of the RNA polymerase II subunit (*rpb2*) gene with primers RPB2150_F and fRPB2-7cR; and a fragment of the translation elongation factor 1-alpha (*tef1*) gene with primers EF1-728F and EF1-2218R following the PCR conditions described in Gardes and Bruns [23], White et al. [24], Jewell and Hsiang [25], Liu et al. [26], and Carbone and Kohn [27], respectively.

The amplicons were sequenced according to the Sanger method by an external service provider. Sequence reads were quality-checked, screened for ambiguous bases, and trimmed at both ends using Geneious Prime 2024 (Biomatters Ltd., Auckland, New Zealand). The sequences were then deposited in NCBI database. The ITS, *rpb2*, and *tub2* sequences from this study were aligned with sequences obtained from GenBank using the MAFFT v7 standard alignment algorithm [28] in Geneious. The sequences of each locus were aligned separately, and alignment gaps were treated as missing character states; all characters were unordered and of equal weight. Maximum likelihood (ML) analyses were performed on the concatenated ITS, *rpb2*, and *tub2* alignment. The ML analysis was executed on the IQ-TREE web server [29] based on the GTR substitution model with gamma distribution rate variation. The alignment and phylogeny are publicly available in 10.5281/zenodo.13305798. The *M. majus* strain was deposited at Westerdijk Fungal Biodiversity Institute (https://wi.knaw.nl/, accessed on 16 September 2024.) under accession number CBS 152328.

The three *B. cinerea* strains used in this study were obtained from the microbial isolate collection of the Food and Wine Research Institute of Eszterházy Károly Catholic University. These three strains (Or11, Or13, and Or33), were previously isolated from grape berries that exhibited the symptoms of bunch rot. Conidia were carefully sprinkled on the surface of a DRBC (Dichloran Rose-Bengal Chloramphenicol) agar plate and incubated at 21 °C in the dark until the emergence of fungal colonies. Conidial suspensions were prepared from the colonies and sequentially streaked two times on the surface of PDA (Potato Dextrose Agar) plates and incubated as described above to obtain monoclonal cultures.

### 2.2. Dual Culture Assays

The possible interactions between *M. majus* strain CBS 152328 and *B. cinerea* Or11, Or13, and Or33 isolates were first studied under four different nutrient conditions in culture because the antagonistic activity of endophytic biocontrol agents may depend on competition for nutrients and other conditions in plant tissues [19]. Co-cultures of *M. majus* and each of the three *B. cinerea* strains included in this study were set up on the following media: (a) mineral salts (1.5 g/L K_2_HPO_4_, 2 g/L KH_2_PO_4_, 5 g/L MgSO_4_) supplemented with 1% (*m/v*) glucose as the sole carbon source and 0.1% (*m/v*) ammonium sulphate as the sole nitrogen source; (b) the same amounts of the salts and glucose as in a) and 10 mM urea as the sole nitrogen source; (c) the same amounts of the salts and ammonium sulphate as in a) and 2% (*v/v*) glycerol as the sole carbon source; and (d) the same amounts of the salts and glycerol as in c) and 10 mM urea as the sole nitrogen source. All media were solidified with 2% (*m/v*) agar. Fungi were inoculated at a 4.5 cm distance in 9 cm diameter plates as mycelial plugs cut from colonies grown on PDA. Co-cultures were incubated at 25 °C in the dark and examined macroscopically and photographed eight days post inoculations (dpi).

For the microscopic investigation of the *M. majus–B. cinerea* interactions, these were co-inoculated as described above on the surface of an autoclaved cellophane sheet placed on 2% (*m/v*) Water Agar (WA). Cultures were incubated at 25 °C in the dark for eight days, then examined under a compound microscope as described below. All co-culturing tests were performed three times.

### 2.3. Phytopathogenicity Tests

Wheat grains cv. IS Carrier were extensively washed with sterile distilled water. Ten grains were placed in a 9 cm diameter plate on autoclaved paper towel discs wetted with sterile distilled water. Three plates prepared in this way were inoculated with *M. majus* and another three with *B. cinerea* strain Or13 by placing five mycelial plugs from PDA cultures of the respective strains on wet paper towel in the plates, near the wheat grains. Three other plates were inoculated with both *M. majus* and *B. cinerea* with five mycelial plugs of each strain placed approx. 1 cm apart from each other. Three control plates were not inoculated with fungal materials.

To carry out phytopathogenicity tests with grapevine, one cm diameter foliar discs were cut from the first fully expanded leaves of five, one-year-old Cabernet Sauvignon cuttings grown in pots. Foliar discs were extensively washed with sterile distilled water, and five of them were placed at the central line of 9 cm diameter plates on top of 2% (*m/v*) Water Agar (WA). Three plates were inoculated with five mycelial plugs of *M. majus* strain CBS 152328; three other plates with *B. cinerea* strain Or13; and another three plates with both fungi, by placing their mycelial plugs at approx. 1 cm distance from the foliar discs. Three control plates were prepared without inoculation.

All plates were incubated at 21±2 °C with a 12h photoperiod under a fluorescent lamp (6500K colour temperature) for seven days. All experiments were performed in triplicate.

### 2.4. Study of the Effects of Molecules Secreted by M. majus on Grapevine Foliar Discs and B. cinerea

Cultures of *M. majus* CBS 152328 were prepared by inoculating 5 mycelial plugs (grown on PDA medium) with one cm diameter in 100 mL Czapek Dox liquid medium. Cultures were incubated in 500 mL flasks, at 25 °C temperature, with 120 rpm shaking, for 6 days. Sterile filtrates were obtained by filtering the cultures first with a cheese filter, then with a membrane with a 0.4 µm pore size.

Effects of the obtained culture filtrate on grapevine leaves were tested by incubating one cm diameter foliar discs (cut from leaves of one-year old potted cuttings of Cabernet Sauvignon) in the filtrate, or in sterile Czapek Dox medium as a control at 21 °C for 24 h. Foliar discs were then subjected to microscopic examination for the visualisation of lignin deposition (see Section 2.5) or placed on the margins of *B. cinerea* Or13 colonies pre-grown on WA at 21 °C for 7 days. These artificially infected discs were incubated for an additional 24 h at 21 °C and photographed. Relative area of the developed necroses were quantified by the use of ImageJ software (version 1.54f). For both the treated and control conditions, five foliar discs were used.

Effects of *M. majus* CBS 152328 culture filtrate on the mycelial growth of *B. cinerea* Or13 were tested by mixing one volume of the filtrate with one volume of two-fold concentrated Czapek Dox agar medium. Mixtures were poured in Petri dishes (9 cm diameter), and three mycelial plugs (grown on PDA) of *B. cinerea* Or13 were inoculated at the margins of the dishes. The procedure was also carried out using sterile Czapek Dox liquid medium instead of the culture filtrate as a control. Cultures were incubated at 21 °C, for 6 days, and radial growth of the *B. cinerea* colonies were measured.

### 2.5. Microscopic Examinations

To investigate the interactions between *M. majus* strain CBS 152328 and *B. cinerea* strains Or11, Or13, and Or33, respectively, on WA, small sections of the cellophane covered by fungal hyphae were cut from the confrontation zone of the dual cultures and examined directly under a compound microscope, or after brief staining with cotton blue in lactophenol. Visualisation of *M. majus* mycelia on inoculated grape leaf discs and wheat grains and germlings was performed by fixing the samples in 70% (*v/v*) ethanol for 2 min, staining with 100 µg/mL Calcofluor White for 2 min, and destaining in distilled water for 2 min. For the investigation of the *M. majus–B. cinerea* mycelial interactions on co-inoculated grape and wheat materials, samples were taken with a cellotape, briefly stained by lactophenol cotton blue, and examined under a compound microscope.

To visualise lignin deposition at the margins of grapevine foliar discs, treated with *M. majus* culture filtrates, or sterile liquid medium (see Section 2.4), samples were stained in 0.5 mM acridine orange solution at room temperature for 5 min, followed by 10 min destaining in distilled water. Discs were briefly dried on a paper towel, placed on a glass slide and examined after covering with a coverslip.

All examinations were conducted using an Olympus BX53F2 microscope (Olympus Corporation, Tokyo, Japan) equipped with differential interference contrast (DIC) optical accessories. Fluorophores were excited by an LED light source (λ = 360–665 nm), T8, 18W, F18W/865 (Osram, Munich, Germany). The blue filter set was used (λex = 360–370 nm, λem = 420–460 nm) for the visualisation of Calcofluor White fluorescence. Acridine orange fluorescence visualised using the green filter set (λex = 470–495 nm, λem = 510–550 nm). Photomicrographs were recorded with a DP74 camera using the CellSens Entry software (version 4.1, Olympus Corporation, Tokyo, Japan).

## 3. Results

### 3.1. Molecular Identification of the M. majus Strain CBS 152328 Isolated from Grapevine

**Figure 1:**
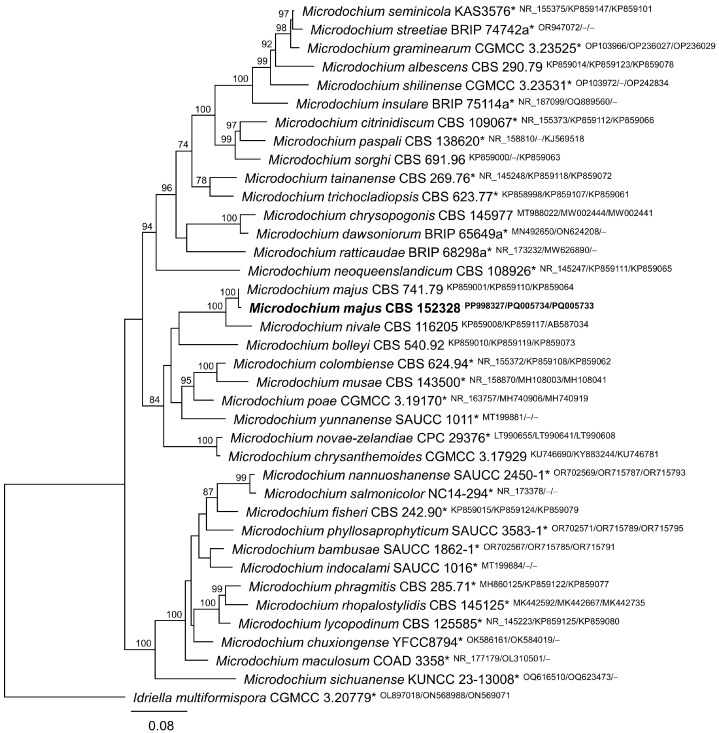
Phylogenetic tree based on the maximum likelihood (ML) analysis of the concatenated alignment of ITS region, rpb2, and tub2 from related species of Microdochium. Idriella multiformispora (ex-type strain CGMCC 3.20779) was used as the outgroup. Bootstrap support values greater than 70% are given at the nodes. GenBank accession numbers are indicated (superscript ITS/rpb2/tub2). The fungal strain isolated and characterised in this study is shown in bold. Ex-type strains are marked by an asterisk (*).

The phylogenetic analysis of three DNA regions (ITS, rpb2, and tub2) sequenced in this study identified the fungal strain CBS 152328 as Microdochium majus (Figure 1). These sequences are available in GenBank (ITS: PP998327; rpb2: PQ005734; tub2: PQ005733). A tef1 fragment was also sequenced in strain CBS 152328 and deposited in GenBank (PQ325511).

### 3.2. Interactions Between M. majus and B. cinerea in Culture

**Figure 2:**
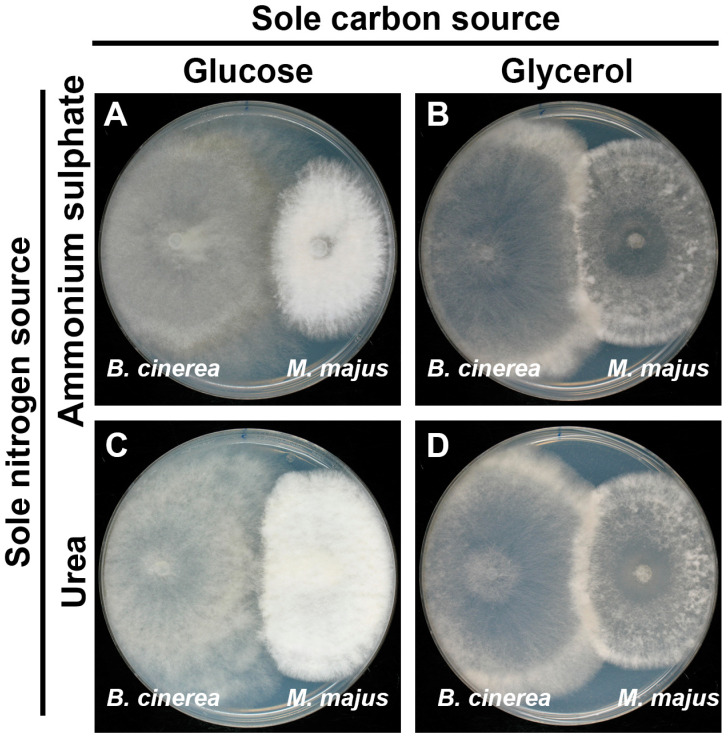
Dual culture assays of *Microdochium majus* and *Botrytis cinerea* Or13 on mineral medium amended with glucose (**A**,**C**) or glycerol (**B**,**D**) as sole carbon sources and ammonium sulphate (**A**,**B**) or urea (**C**,**D**) as sole nitrogen sources. Colonies were grown at 25 °C. Photographs were taken at 8 dpi.

Dual cultures of the two fungi were performed on four different media to test whether there were any signs of antagonism between them. These preliminary experiments revealed that the *M. majus* colonies had overgrown the colonies of *B. cinerea* in all plates with glycerol, regardless of the available nitrogen source (ammonium sulphate or urea). Overgrowth of *M. majus* on *B. cinerea* colonies was inhibited in the presence of glucose (Figure 2A,C). It was also noted that both fungal strains produced a much thinner mycelium on glycerol (Figure 2B,D). This observation may suggest that these fungi have a poor ability to utilise glycerol as a carbon source.

**Figure 3:**
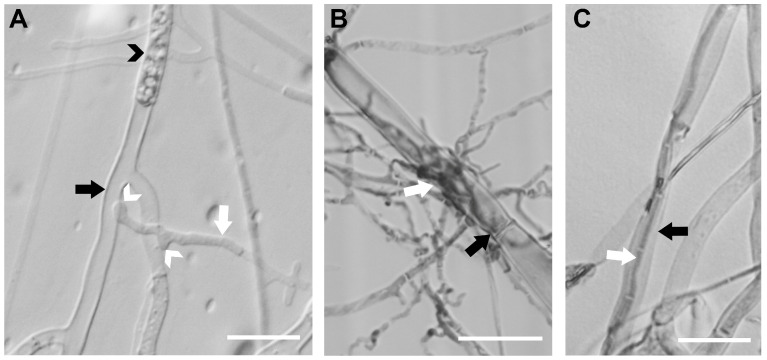
Photomicrographs of the interacting hyphae of *Microdochium majus* and *Botrytis cinerea* grown on Water Agar at 25 °C. (**A**) DIC photomicrograph taken at 5 dpi. (**B**) Bright field photomicrograph of colonised host hypha taken at 8 dpi, after staining with cotton blue in lactophenol. (**C**) Intracellular growth of a hypha of *M. majus* inside a hypha of *B. cinerea*, photographed at 8 dpi, after staining with cotton blue in lactophenol. Black arrows indicate *B. cinerea* hyphae; white arrows point to *M. majus* hyphae. Black arrowheads indicate condensed cytoplasm; white arrowheads point to papilla-like structures. Scale bars represent 20 µm.

Microscopic examination of the interacting hyphae of *B. cinerea* and *M. majus* on sterile cellophane placed on WA detected structures typical of mycoparasitism (Figure 3). Their hyphae were readily distinguished under the microscope, as *B. cinerea* hyphae are much thicker, ~4–8 µm, compared to the hyphae of *M. majus* that are only ~1–3 µm. Attachment of the hyphal tips and papilla formation on the *B. cinerea* host surface (Figure 3A, white arrowheads) was detected as early as 5 dpi. The attacked hyphal compartments were often empty (Figure 3A, black arrow), and the cytoplasm of the surrounding mycohost cells was sometimes condensed, indicating the death of those cells (Figure 3A, black arrowhead). The massive colonisation of the mycohost hyphae by *M. majus* was observed at 8 dpi (Figure 3B). Intracellular growth of *M. majus* in the *B. cinerea* hyphae was also detected at this later stage of the interaction (Figure 3C).

### 3.3. Effects of M. majus and B. cinerea Inoculations on Germinating Wheat Grains and Grape Leaf Discs

**Figure 4:**
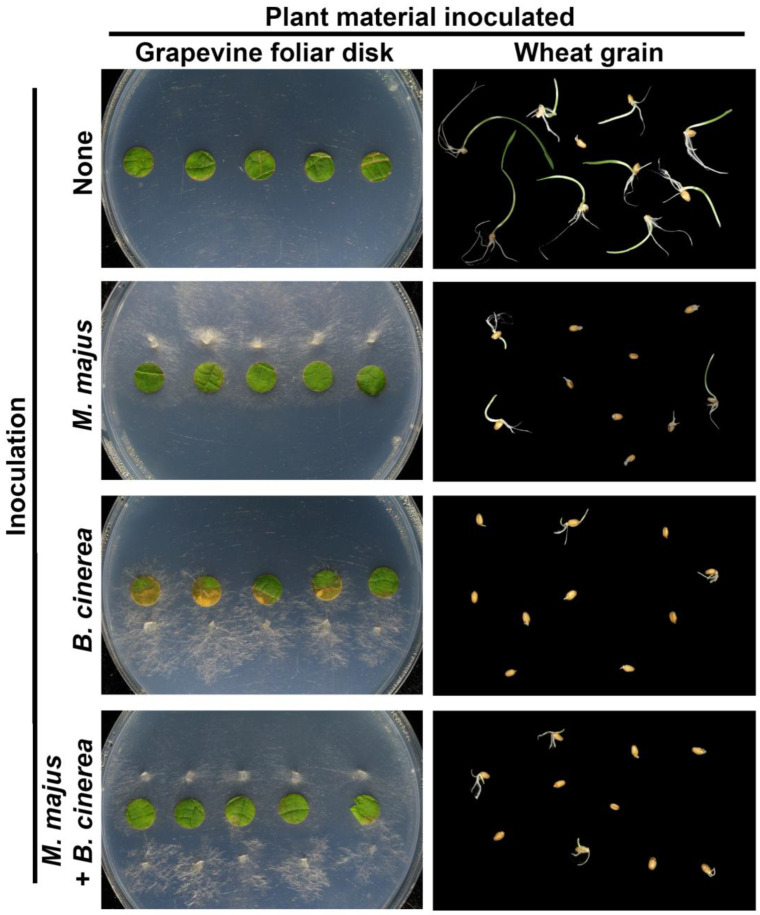
Photographs of grapevine foliar discs on Water Agar (4 dpi) and wheat grains germinated on a wet paper towel (6 dpi). Plant materials were left uninoculated or inoculated with *Microdochium majus*, *Botrytis cinerea*, and with both.

**Figure 5:**
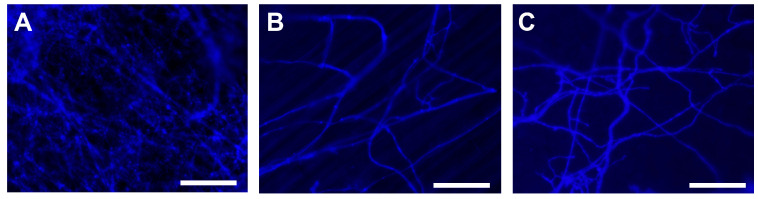
Fluorescent photomicrographs of *M. majus* mycelia, stained with Calcofluor White, growing on wheat grain (**A**, 8 dpi), on the stem of a germinated wheat (**B**, 8 dpi), and on a grapevine foliar disc (**C**, 6 dpi). Scale bars represent 100 µm.

Visual examination of the grapevine leaf discs at 4 dpi, and the wheat grains and plantlets at 6 dpi, respectively, indicated that *M. majus* inhibited the germination of wheat grains, but it had no negative impact on the grape foliar discs. The *B. cinerea* strain Or13 has also inhibited wheat germination and damaged the grape leaf discs as well (Figure 4). Fluorescent microscopy revealed that the mycelia of *M. majus* colonised all inoculated plant surfaces, i.e., wheat grains, the stems of germinated wheat plantlets, and grapevine foliar discs as well (Figure 5).

### 3.4. Hyphal Interactions of M. majus and B. cinerea on Grapevine and Wheat Tissues

When *M. majus* was inoculated in combination with *B. cinerea* on grapevine foliar discs, visual examinations indicated that *M. majus* inhibited the development of plant necroses and discolorations caused by *B. cinerea* 4 dpi. On germinating wheat grains, *M. majus* did not have an impact on the damage caused by *B. cinerea* through the inhibition of wheat germination (Figure 4).

**Figure 6:**
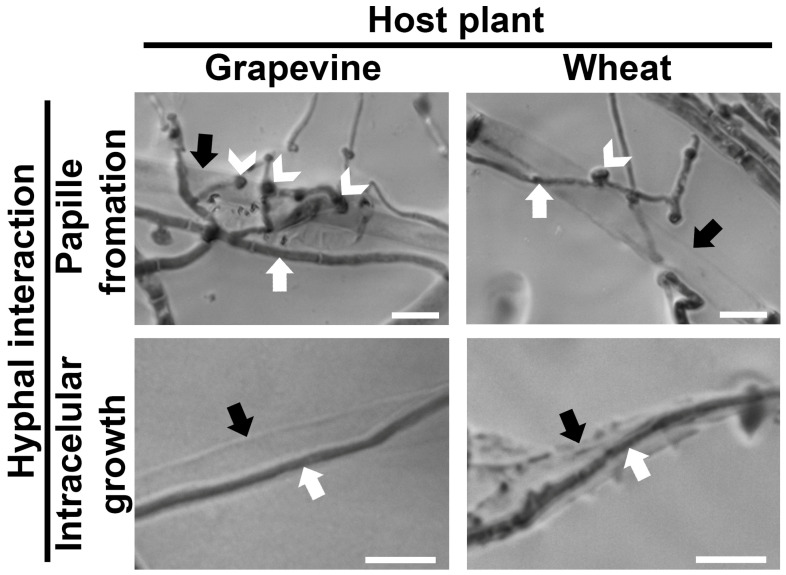
Photomicrographs of the interacting hyphae of *M. majus* and *B. cinerea* at 6 dpi following co-inoculations on opposite sides of grapevine leaf discs and 8 dpi following co-inoculations on opposite sides of germinating wheat grains placed on Water Agar. Photographs were taken after staining with cotton blue in lactophenol. Black arrows indicate *B. cinerea* hyphae; white arrows point to *M. majus* hyphae. White arrowheads indicate papilla-like structures. Scale bars represent 10 µm.

Microscopic studies revealed that the interactions between the hyphae of *M. majus* and *B. cinerea* on co-inoculated grape leaf discs and germinated wheat plantlets, and also on WA, before *B. cinerea* hyphae that emerged from mycelial plugs reached the plant tissues, led to the development of microscopic structures characteristic of mycoparasitic interactions (Figure 6). These were similar to the structures observed in dual cultures (Figure 3). The formation of papilla-like structures, as well as the intracellular growth of *M. majus*, was observed when it established contact with *B. cinerea* hyphae (Figure 6).

### 3.5. Effects of M. majus Secreted Molecules on Grapevine and B. cinerea

**Figure 7:**
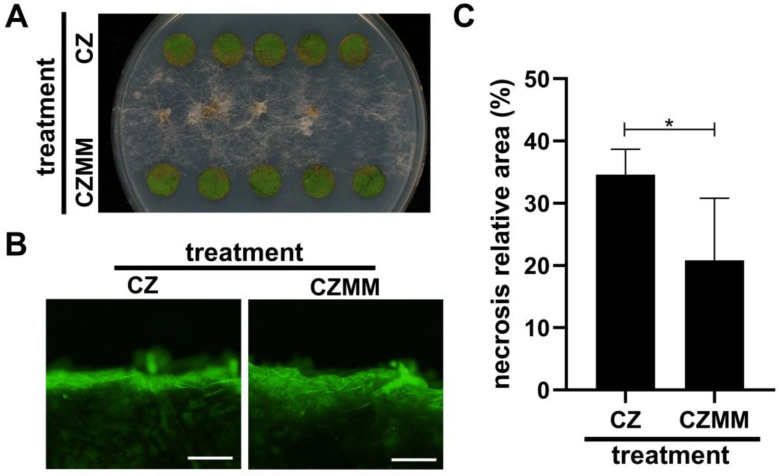
Effects of 24h treatment with Czapek Dox liquid medium (CZ), or with the culture filtrate of *M. majus* (CZMM; Czapek Dox liquid medium, 25 °C, 6 dpi, 120 rpm shaking) on grapevine foliar discs. (**A**) Photograph of leaf discs after one-day incubation near *B. cinerea* Or13 colonies pre-grown on WA. (**B**) Fluorescent photomicrographs of foliar disc margins, stained with acridine orange. Scale bars represent 100 µm. (**C**) Percental relative areas of necrosis developed on foliar discs inoculated with *B. cinerea* Or13 (analysis of Figure 7A). Statistical analysis was performed using Student’s *t*-test. Asterisk marks the significance of the difference (* *p* < 0.05).

Pretreatment of grapevine foliar discs with the culture filtrate of *M. majus* CBS 152328 resulted in decreased susceptibility towards *B. cinerea* infection relative to a control treatment with a sterile liquid medium (Figure 7A). The quantification of the percental relative necrotized area of the artificially infected foliar discs reinforced the disease-preventing capability of the secreted molecules of *M. majus*, which significantly decreased the damage caused by *B. cinerea* (Figure 7C). The decreased susceptibility of the leaf tissues treated with the culture filtrate was in accordance with the observed increased lignification of the damaged margins of uninoculated leaf discs, suggested by their more explicit staining by acridine orange (Figure 7B).

**Figure 8:**
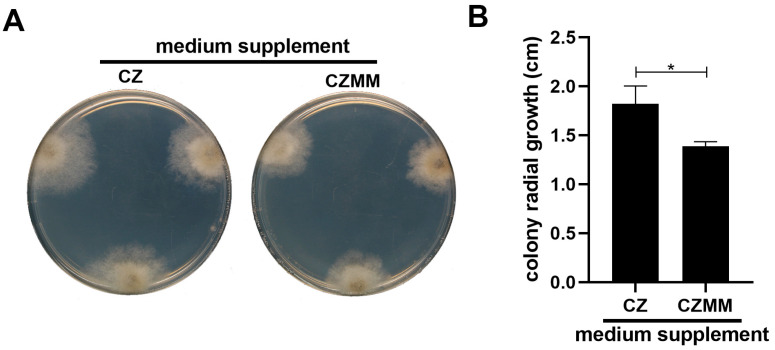
Effects of Czapek Dox liquid medium (CZ), or the culture filtrate of *M. majus* (CZMM; Czapek Dox liquid medium, 25 °C, 6 dpi, 120 rpm shaking) on the growth *B. cinerea*. (**A**) Photographs of *B. cinerea* Or13 colonies growing on Czapek Dox agar amended with 50% (*v/v*) CZ or CMM (21 °C, 6 dpi). (**B**) Radial growth *B. cinerea* Or13 in the presence of 50% (*v/v*) CZ or CMM (analysis of Figure 8A). Statistical analysis was performed using Student’s *t*-test. The asterisk marks the significance of the difference (* *p* < 0.05).

The secreted molecules of *M. majus* CBS 152328 also showed a direct negative effect on *B. cinerea* growth. Inoculating *B. cinerea* on a medium amended with 50% (*v/v*) culture filtrate of *M. majus* resulted in the development of smaller colonies relative to the control (Figure 8A). While this difference was marginal, the inhibitory effect of *M. majus* culture filtrate proved to be significant according to the statistical analysis of the *B. cinerea* radial growth (Figure 8B).

## 4. Discussion

The isolation of a *M. majus* strain from grapevine was surprising, as this species, similar to other *Microdochium* spp., is mainly known as a pathogen of small-grain cereals and grasses [2,3,4]. To our knowledge, this is the first report of *M. majus* in a dicot host. Reports of *Microdochium* spp. from dicot plant tissues are sporadic, and the species-specific identification of the detected *Microdochium* strains was not achieved in these studies due to the limitations of the metabarcoding techniques or other technical issues [10,11,12]. The isolation of *M. majus* from an asymptomatic grapevine cane and lack of pathogenicity to grape leaf discs as revealed in this work may indicate its endophytic lifestyle in grapevine tissues. These unexpected findings triggered the present work on its possible role as a yet unnoticed antagonist of fungal pathogens that occur in the same environment.

*Microdochium* species have been recognised as fusarium-like fungi [2], and some *Fusarium* spp., in addition to their well-known role in cereal diseases [2,3,4], have also been recorded as necrotrophic mycoparasites of *Rhizoctonia solani* [30], *Plasmopara viticola* [31] and *B. cinerea* [32]. This work revealed that the *M. majus* strain CBS 152328 from grapevine is also a necrotrophic mycoparasite of *B. cinerea*. This was demonstrated in the confrontation zones of dual cultures of *M. majus* and *B. cinerea* on WA, thus under nutrient-deprived conditions (Figure 3), and also on the surface of co-inoculated grape leaf discs and germinated wheat tissues (Figure 6). Inhibition zones that might have indicated antibiosis, i.e., the production of antifungal metabolites by *M. majus*, were not observed in dual cultures. This may indicate that the growth of *B. cinerea* was suppressed by *M. majus* in the confrontation zones solely through mycoparasitism in dual cultures (Figure 2).

Earlier studies indicated that mycoparasitic behaviour that is similar to those observed in this study may sometimes be linked to ‘starvation’, i.e., nutrient-deprived conditions. The expression profile of a mitogen-activated protein kinase (MAPK) gene in *Stachybotrys elegans* was similar during carbon-poor conditions and its mycoparasitic interaction with *Rhizoctonia solani* that was considered a carbon-rich environment [33]. Mycoparasitism-related genes were downregulated in *Trichoderma harzianum* by glucose [34,35]. In this study, dual culture assays revealed that overgrowth of *B. cinerea* colonies by *M. majus* occurred on mineral media supplemented with glycerol and either urea or ammonium sulphate as the sole nitrogen source, but not on any media with glucose as the carbon source. *Microdochium majus* acted as a mycoparasite of *B. cinerea* on media with glycerol and also on WA and on the surface of grape leaf discs and germinated wheat tissues. This may indicate that its mycoparasitic activity is mainly expressed in nutrient-deprived conditions.

Phytopathogenicity tests with *M. majus* indicated that the fungus colonised both grape leaf discs and germinated wheat but only damaged wheat by inhibiting its germination. When the tested plant materials were co-inoculated with both *M. majus* and *B. cinerea* on WA, with mycelial plugs placed on the opposite sides of the plant samples (Figure 4), *M. majus* parasitized the hyphae of *B. cinerea* on the surface of both grape and wheat tissues (Figure 6). The presence of hyphal interactions characteristic of mycoparasitism on the colonised wheat grains suggests that the mycoparasitic behaviour of *M. majus* was expressed even in the presence of a susceptible plant, allowing the fungus to obtain nutrients simultaneously from different hosts. *M. majus* was also able to parasitize and suppress *B. cinerea* on grape leaf discs, and also on WA around the foliar discs. However, it did not have a negative impact on grape leaves and even protected this plant host from the damage that could have been caused by *B. cinerea*.

While the mycoparasitic behaviour of *M. majus* on *B. cinerea* mycohost was clearly demonstrated in vitro, as well as on grapevine, some additional mechanisms may also contribute to its disease-preventing effect on tissues of this plant host. Treatment of the grape foliar discs with the culture filtrate of *M. majus* decreased their susceptibility towards the *B. cinerea* infection (Figure 7A). However, its efficiency was lower compared to the direct inoculation of *M. majus*, which completely prevented any damage that would be caused by the co-inoculated *B. cinerea* (Figure 4). The increased disease resistance of foliar discs in response to the treatment with the *M. majus* culture filtrate may have originated from the effect of ‘microbe-associated molecular patterns’ (MAMPs) eliciting defence responses in plants [36]. Reinforcing this possibility, increased lignification was observed at the damaged margins of grape discs treated with *M. majus* culture filtrate and left uninoculated (Figure 7B). The lignification may directly be responsible for the increased resistance of grape foliar discs since it is a general plant response against *B. cinerea* infections [37,38]. An inhibitory effect of *M. majus* culture filtrate on the in vitro growth of *B. cinerea* was also observed (Figure 8A); however, its impact was very low (Figure 8B). In conclusion, while *M. majus* expressed an antibiotic effect against *B. cinerea*, as well as an elicitor effect on grapevine leaf tissues, their low efficiency suggests that the main mode of action of *M. majus* against *B. cinerea* is mycoparasitism.

A comprehensive review of all fungal species known as mycoparasites did not list any *Microdochium* species [39]. This present work appears to be the first report of the mycoparasitic behaviour of *M. majus*, in addition to its first isolation from a dicot host. This lifestyle of a fungus that is mainly known as a cereal pathogen could be further investigated with molecular tools in future studies.

## Data Availability

Nucleic acid sequences were uploaded to GenBank. Any other research data are presented in this study.
